# Erratum to “Exploring the universal healthy human gut microbiota around the World”

**DOI:** 10.34133/csbj.0116

**Published:** 2026-06-18

**Authors:** Samuel Piquer-Esteban, Susana Ruiz-Ruiz, Vicente Arnau, Wladimiro Diaz, Andrés Moya

**Affiliations:** ^1^ Institute of Integrative Systems Biology (I2Sysbio), University of Valencia and Consejo Superior de Investigaciones Científicas (CSIC), Valencia 46980, Spain.; ^2^ Genomic and Health Area, Foundation for the Promotion of Health and Biomedical Research of Valencia Region, Valencia 46020, Spain.; ^3^ Centro de Investigación Biomedica en Red en Epidemiologia y Salud Publica (CIBEResp), Madrid 28029, Spain.

In the Research article entitled “Exploring the universal healthy human gut microbiota around the World”, the authors noticed an omission in the funding information [1]. Specifically, it needs to be noted that the project PID2019-105969GB-I00 was funded by MICIU/AEI /10.13039/501100011033. The Acknowledgments section has now been updated in the original record and is also provided below.

The authors apologize for this error.
